# Integrated analysis of the M2 macrophage-related signature associated with prognosis in ovarian cancer

**DOI:** 10.3389/fonc.2022.986885

**Published:** 2022-08-26

**Authors:** Caijiao Peng, Licheng Li, Guangxia Luo, Shanmei Tan, Ruming Xia, Lanjuan Zeng

**Affiliations:** ^1^ Department of Gynecological Oncology, The Fourth Affiliated Hospital of Jishou University, Huaihua, China; ^2^ Department of Gynecological Oncology, the First People’s Hospital of Huaihua, Huaihua, China; ^3^ Clinical Medical College, Guizhou Medical University, Guiyang, China

**Keywords:** macrophage, prognosis, ovarian cancer, pathway, immune

## Abstract

**Background:**

M2 macrophages play an important role in cancer development. However, the underlying biological fator affecting M2 macrophages infiltration in ovarian cancer (OV) has not been elucidated.

**Methods:**

R software v 4.0.0 was used for all the analysis. The expression profile and clinical information of OV patients enrolled in this study were all downloaded from The Cancer Genome Atlas and Gene Expression Omnibus databases.

**Results:**

The CIBERSORT algorithm was used to quantify the M2 macrophage infiltration in OV tissue, which was found a risk factor for patients survival. Based on the limma package, a total of 196 DEGs were identified between OV patients with high and low M2 macrophage infiltration, which were defined as M2 macrophages related genes. Finally, the genes PTGFR, LILRA2 and KCNA1 were identified for prognosis model construction, which showed a great prediction efficiency in both training and validation cohorts (Training cohort, 1-year AUC = 0.661, 3-year AUC = 0.682, 8-year AUC = 0.846; Validation cohort, 1-year AUC = 0.642, 3-year AUC = 0.716, 5-year AUC = 0.741). Clinical correlation showed that the riskscore was associated with the worse clinical features. Pathway enrichment analysis showed that in high risk patients, the pathway of epithelial-mesenchymal transition (EMT), TNF-α signaling *via* NFKB, IL2/STAT5 signaling, apical junction, inflammatory response, KRAS signaling, myogenesis were activated. Moreover, we found that the PTGFR, LILRA2 and KCNA1 were all positively correlated with M2 macrophage infiltration and PTGFR was significantly associated with the pathway of autophagy regulation. Moreover, we found that the low risk patients might be more sensitive to cisplatin, while high risk patient might be more sensitive to axitinib, bexarotene, bortezomib, nilotinib, pazopanib.

**Conclusions:**

In this study, we identified the genes associated with M2 macrophage infiltration and developed a model that could effectively predict the prognosis of OV patients.

## Introduction

Globally, ovarian cancer (OV) is the seventh most common and deadliest gynecologic malignancy, with 313,959 new cases and 207,252 deaths in 2020 (1). Due to the susceptibility to recurrence, metastasis and drug resistance development of OV, the 5-year overall survival (OS) rate of most patients is less than 30% (2). Worse still, due to the location of the ovaries deep within the pelvis, ovarian cancer is difficult to detect at an early stage (3). Although patients may initially respond well to the conventional treatment including surgery and paclitaxel/carboplatin combination chemotherapy, recurrence occurs in up to 80% of patients, with 20%–30% relapsing or progressing within six months (4). In recent years, high-throughput sequencing technology and transcriptomic research have enabled researchers to uncover numerous key driver genes, and many novel treatment methods have been discovered. However, a significant advancement in the prognosis of ovarian cancer cells has not been observed. Therefore, precise and individualized predictive biomarkers, especially those affecting the immune microenvironment, are urgently needed for precision therapy of ovarian cancer.

Macrophages can regulate the immune response against pathogens and is an important component in the tumor microenvironment (5). In the tumor microenvironment, the crosstalk of tumor-associated macrophages (TAMs) with tumor cells could significantly affect cancer progression (6). Generally, macrophages could be divided into two subsets: M1 macrophages (immune-stimulatory macrophages) and M2 macrophages (immune-regulatory macrophages). In most cancer, M2 macrophages might play a cancer-promoting role. For instance, Zhao et al. found that tumor-derived exosomal miR-934 could induce macrophage M2 polarization to promote liver metastasis of colon cancer (7). Chen et al. indicated that tumor-recruited M2 macrophages could facilitate gastric and breast cancer metastasis through M2 macrophage-secreted CHI3L1 protein (8). Xu et al. revealed that Astragaloside IV could inhibit lung cancer progression and metastasis by modulating macrophage polarization through AMPK signaling (9). Also, Zhang et al. demonstrated that tumoral NOX4 could recruit M2 tumor-associated macrophages by ROS/PI3K signaling-dependent various cytokine production to promote NSCLC growth (10). In OV, Zeng et al. found that the EGF secreted by M2 macrophages could promote epithelial OV metastasis through EGFR-ERK signaling (11). An et al. found that miR-21 could modulate the polarization of macrophages and increases the effects of M2 macrophages on promoting the chemoresistance of OV (12). The investigation of underlying biological factors affecting M2 macrophage infiltration in OV is necessary and meaningful.

In our study, we systematically analyzed the genes associated with M2 macrophage infiltration in OV. PPI network was constructed to explore the underlying interaction of these genes. A prognosis model based on PTGFR, LILRA2 and KCNA1 was established, which showed great prediction efficiency in both training and validation cohorts. A nomogram was constructed for a better application in the clinical. Then, clinical correlation and pathway enrichment analysis were performed to explore the underlying differences between high and low risk groups. Meanwhile, the model gene PTGFR, LILRA2 and KCNA1 were further explored for future studies.

## Methods

### Open-accessed data acquisition

The expression profile and clinical information of OV patients were obtained from The Cancer Genome Atlas (TCGA, https://portal.gdc.cancer.gov/) and Gene Expression Omnibus (GEO, https://www.ncbi.nlm.nih.gov/, GEO datasets) databases. In the TCGA database, the data were downloaded from the TCGA-OV project. The transcriptional profiling data was “TPM” form and clinical information was “bcr xml” form, which were collated using the author’s code. GSE26712 (GPL96, Affymetrix Human Genome U133A Array), GSE51088 (GPL7264, Agilent-012097 Human 1A Microarray) and GSE53963 (GPL6480, Agilent-014850 Whole Human Genome Microarray) were downloaded from GEO database. The patients with complete gene expression profile and clinical information were enrolled in our study. The data standardization process was carried out before analysis, including data alignment, missing value processing, probe annotation. Sva package was used to reduce the batch effect in different independent cohorts.

### Differentially expressed genes analysis and immune infiltration quantification

CIBERSORT algorithm was used to quantify the proportions of 22 immune cells, including M2 macrophages. Differentially expressed genes (DEGs) analysis was performed using the limma package with the threshold of |logFC| > 1 and P.value < 0.05 (13).

### Protein-protein interaction network

PPI network was established based on the STRING database with input genes. Detailed, the nodes with high confidence (0.700) were identified (14). The cytoscape 3.7.2 was used for the visualization of PPI network. The cytoHubba plug-in was used to identify the hub nodes. The ClueGO plug-in was used to perform gene ontology (GO) analysis for the network nodes, in which the enrichment terms was “GO, Biological Process” (15). With ClueGO, GO terms and Kyoto Encyclopedia of Genes and Genomes (KEGG)/BioCarta pathways are integrated to create a functionally arranged network of GO/pathway terms.

### Prognosis model construction

For the DEGs identified between high and low M2 macrophages, univariate Cox regression analysis was performed to screen the prognosis-related genes with threshold of P < 0.05. Then, LASSO regression and multivariate Cox regression analysis were performed for prognosis model construction with the formula of “Riskscore = Gene A * Coef A + Gene B * Coef B + Gene C * Coef C + … + Gene N * Coef N” (16). Kaplan-Meier survival and Receiver Operating Characteristic (ROC) curves were used to evaluate the prediction efficiency of our model.

### Nomogram plot and calibration curve

A nomogram was established with the combination of riskscore and clinical features for a better application of the model in clinical (17). Calibration curve was used to compare the difference between nomogram predicted survival and actual survival.

### Pathway enrichment analysis

Pathway enrichment analysis was performed using the Gene Set Enrichment Analysis (GSEA), aimed to explore the underlying biological differences between high and low risk patients (18). In detail, the reference pathway set was h.all.v7.0.symbols.gmt, c5.go.v7.5.1.symbols.gmt and c2.cp.kegg.v7.5.1.symbols.gmt. The pathway terms meeting the criteria of |NES|> 1 and P-value < 0.05 were considered as significant.

### Immunotherapy and chemotherapy sensibility

Immunotherapy evaluation was performed using the Tumor Immune Dysfunction and Exclusion (TIDE) algorithm (19). Chemotherapy sensibility was evaluated based on the Genomics of Drug Sensitivity in Cancer (GDSC) database (20).

### Single-cell analysis

The expression level of identified genes at the single-cell level was analyzed through the Tumor Immune Single-cell Hub (TISCH) database (http://tisch.comp-genomics.org/home/), which provides detailed cell-type annotation at the single-cell level, enabling the exploration of tumor microenvironment across different cancer types.

### Statistical analysis

All the statistical analysis were carried out using the R software v 4.0.0. The statistical P-value was two-sided and < 0.05 was regarded as statistically significant. For the data with the normal distribution, the students T-test was used for statistical analysis. For the data with non-normal distribution, the Wilcoxon rank-sum test was used.

## Results

### Quantification of M2 macrophages

**Figure 1:**
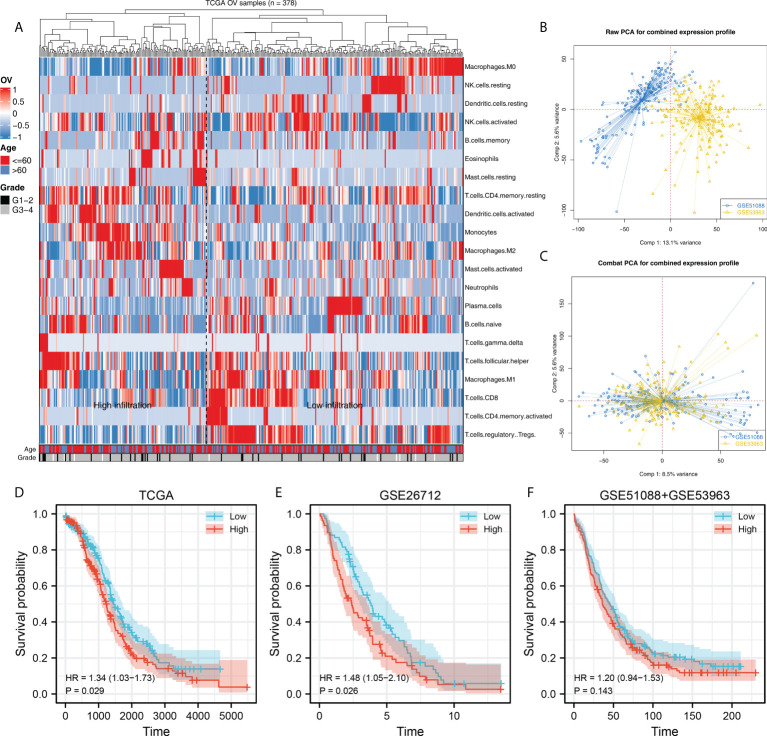
Quantification of the M2 macrophaegs in OV. **(A)** CIBERSORT algorithm was used to quantify the immune cell infiltration in TCGA-OV project; **(B)** GSE51088 and GSE53963 were selected for further analysis; **(C)** Sva package was used to combine the data of GSE51088 and GSE53963; **(D–F)** Kaplan-Meier survival was performed to explore the prognosis role of M2 macrophages in different groups.

The flow chart of the whole study was shown in **Figure S1**. Firstly, 22 immune cells, including M2 macrophages, were quantified through the CIBERSORT algorithm, which was shown in **Figure 1A**. A remarkable batch effect was noticed between the GSE51088 and GSE53963 (**Figure 1B**). Sva package was used for data combination and batch effect reduction. After that, a significantly decreased batch effect between GSE51088 and GSE53963 was observed (**Figure 1C**). Kaplan-Meier survival curves showed that the OV patients with higher M2 macrophage infiltration might have a worse overall survival (**Figures 1D–F**, TCGA, HR = 1.34, P = 0.029; GSE26712, HR = 1.48, P = 0.026; GSE51088 + GSE53963, HR = 1.20, P = 0.143). Moreover, we explored the correlation between M2 macrophages and other immune cells. The result showed that M2 macrophages was positively correlated with the monocytes and neutrophils, yet negatively correlated with the naive B cells, plasma cells, follicular helper T cells, Tregs, activated NK cells, M0 macrophages and activated dendritic cells (**Figure S2**).

### PPI network

**Figure 2:**
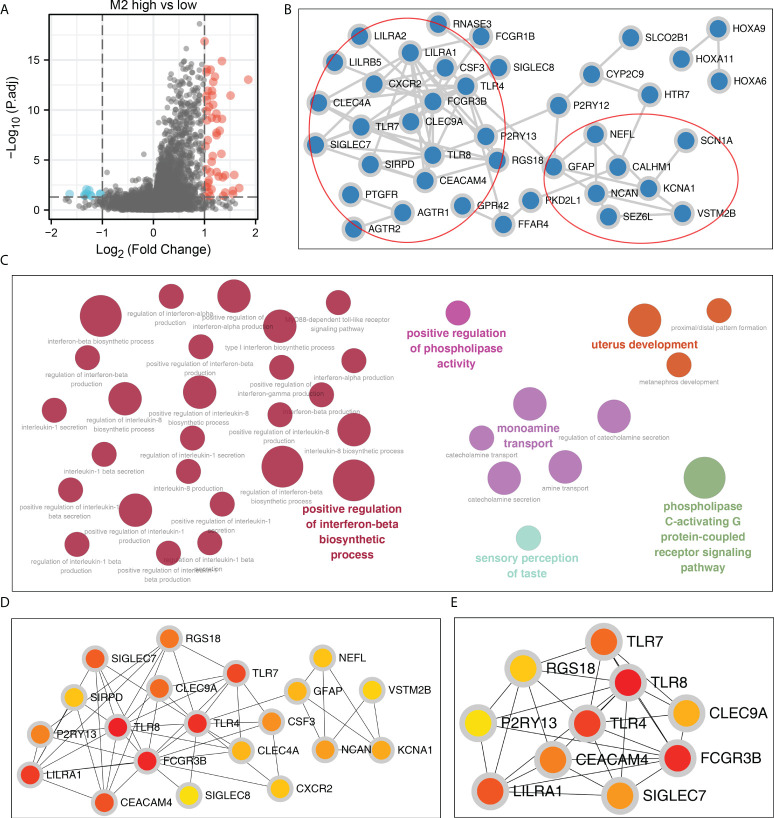
Construction of the PPI network. **(A)** DEGs were identified between high and low M2 macrophages patients using the limma package with the threshold of |logFC| > 1 and P.value < 0.05; **(B)** The PPI network of the DEGs; **(C)** ClueGO analysis of nodes; **(D, E)** Top 20 and 10 important nodes of the PPI network based on the cytohubba plug in.

A total of 196 DEGs were identified between OV patients with high and low M2 macrophages infiltration, which were defined as M2 macrophages related genes (**Figure 2A**). Based on the STRING database, the PPI network of these DEGs was constructed, which have two modules (**Figure 2B**). ClueGO results showed that these DEGs were mainly involved in positive regulation of interferon (IFN)-β biosynthetic process, positive regulation of phospholipase activity, monoamine transport, uterus development, sensory perception of taste, phospholipase C-activating G protein-coupled receptor (**Figure 2C**). The top 20 important nodes of PPI network were shown in **Figure 2D**. The top ten nodes were shown in **Figure 2E**, including TLR7, TLR4, TLR8, RGS18, P2RY13, CEACAM4, LILRA1, SIGLEC7, FCGR3B and CLEC9A.

### Prognosis model construction

**Figure 3:**
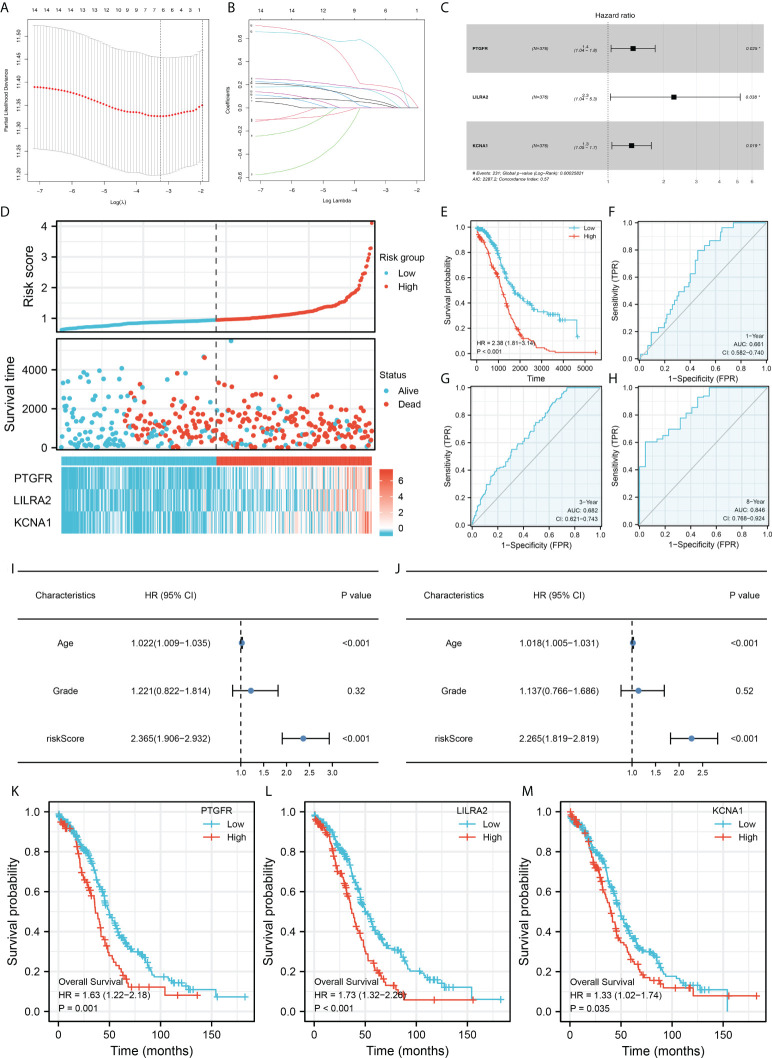
Construction of the prognosis model. **(A, B)** LASSO regression was used for dimensionality reduction; **(C)** Multivariate Cox regression analysis identified three genes for prognosis model construction, including PTGFR, LILRA2 and KCNA1; **(D)** Overview of the model in training cohorts; **(E)** Kaplan-Meier survival curves of patients in high and low risk group in the training group; **(F–H)** ROC curves of the 1-, 3- and 8-year survival; **(I–J)** Univariate **(I)** and multivariate **(J)** analysis were performed to evaluate the independence of our model; **(K–M)** Kaplan-Meier survival was performed to explore the prognosis role of model genes in overall survival.

Firstly, the TCGA database was selected as the training cohort and the combinedGSE cohort was selected as the validation cohort. For the M2 macrophage-related gene mentioned above, the univariate Cox regression analysis and firstly performed to identify the prognosis-related genes (**Table 1**). Next, LASSO regression analysis was used for dimensionality reduction (**Figures 3A, B**). Finally, the genes PTGFR, LILRA2, KCNA1 were identified through multivariate Cox regression analysis for model construction (**Figure 3C**). The riskscore was calculated with the formula of “Riskscore = PTGFR * 0.312 + LILRA2 * 0.819 + KCNA1 * 0.293”. According to the median riskscore, patients were divided into high and low risk groups. In the training cohort, a higher percentage of dead cases was observed in the high risk group (**Figure 3D**). Kaplan-Meier survival curve showed that the patients in high risk group tend to have a worse prognosis compared to the low risk patients (**Figure 3E**). ROC curve showed that our model had a good prediction efficiency in patients survival (**Figures 3F-H**, 1-year AUC = 0.661, 3-year AUC = 0.682, 8-year AUC = 0.846). Univariate and multivariate analysis showed that our model was an independent prognosis factor (**Figures 3I–J**, univariate, HR = 2.365, P < 0.001; multivariate, HR = 2.265, P < 0.001). Kaplan-Meier survival curve showed that the three model genes PTGFR, LILRA2 and KCNA1 were all risk factors for patients prognosis (**Figures 3K– M**, PTGFR, HR = 1.63, P = 0.001; LILRA2, HR = 1.73, P < 0.001; KCNA1, HR = 1.33, P = 0.035).

**Table 1:** Genes meeting the criteria of univariate Cox regression.

id	HR	HR.95L	HR.95H	P value
LILRA2	1.44	1.18	1.76	<0.01
PTGFR	1.56	1.21	2.01	<0.01
CXCR2	1.47	1.18	1.84	<0.01
FRMD7	3.26	1.46	7.26	<0.01
NEFL	1.31	1.08	1.58	0.01
HTR7	1.70	1.15	2.50	0.01
VENTX	1.28	1.05	1.57	0.01
SLITRK3	1.59	1.10	2.32	0.01
P2RY12	1.26	1.04	1.53	0.02
ATP8B4	1.50	1.07	2.11	0.02
SLCO2B1	1.12	1.02	1.24	0.02
FCGR3B	1.23	1.03	1.46	0.02
LILRA1	1.30	1.02	1.64	0.03
MRO	1.46	1.03	2.06	0.03
KCNA1	1.45	1.01	2.08	0.04
HOXA9	1.11	1.00	1.23	0.04

### Validation of the prognosis model

**Figure 4:**
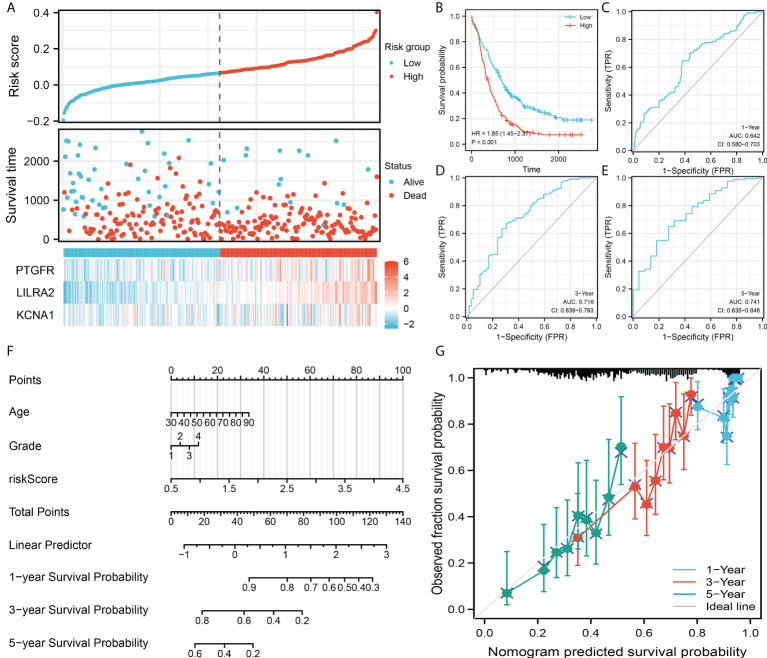
Model validation and nomogram. **(A)** Overview of the model in the validation group; **(B)** Kaplan-Meier survival curves of patients in high and low risk group in the validation group; **(C–E)** ROC curves of the 1-, 3- and 5-year survival; **(F)** A nomogram was established by combining the riskscore and clinical features; **(G)** Calibration curves indicated a good fitting degree between the nomogram predicted survival and the actual survival.

The same trend was also observed in the validation cohort (**Figure 4A**). Kaplan-Meier survival curve indicated that the patients in high risk group might have shorter OS (**Figure 4B**). ROC curves showed that the prediction efficiency of our model in the validation cohort is still satisfactory (**Figures 4C–E**, 1-year AUC = 0.642, 3-year AUC = 0.716, 5-year AUC = 0.741). Then, a nomogram was established for a better clinical application in the clinical (**Figure 4F**). Calibration curves indicated a high-fit between the nomogram predicted survival and actual survival of 1-, 3- and 5-years (**Figure 4G**).

### Clinical correlation and pathway enrichment analysis

**Figure 5:**
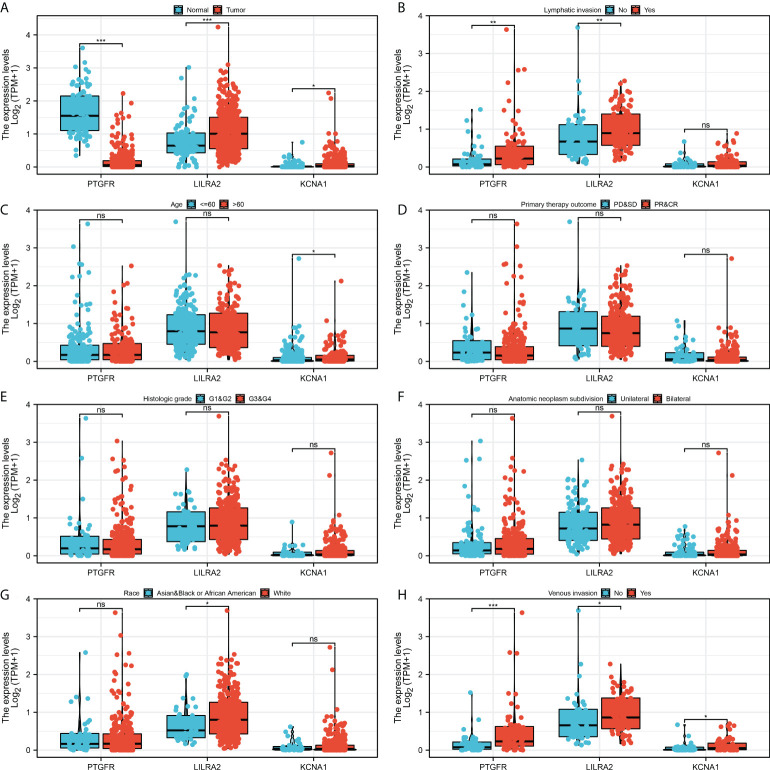
Clinical correlation of the model genes. **(A)** The expression level of model genes in normal and tumor tissue, * = P < 0.05, *** = P <0.001; **(B)** The expression level of model genes in patients with or without lymphatic invasion, ns = P > 0.05, ** = P < 0.01; **(C)** The expression level of model genes in <= 60 and > 60 years old patients, ns = P > 0.05, * = P < 0.05; **(D)** The expression level of model genes in patients with different primary therapy outcomes, ns = P > 0.05; **(E)** The expression level of model genes in patients with different histologic grade, ns = P > 0.05; **(F)** The expression level of model genes in patients with unilateral and bilateral anatomic neoplasm subdivision, ns = P > 0.05; **(G)** The expression level of model genes in different race patients, ns = P > 0.05, * = P < 0.05; **(H)** The expression level of model genes in patients with or without venous invasion, * = P < 0.05, *** = P <0.001.

**Figure 6:**
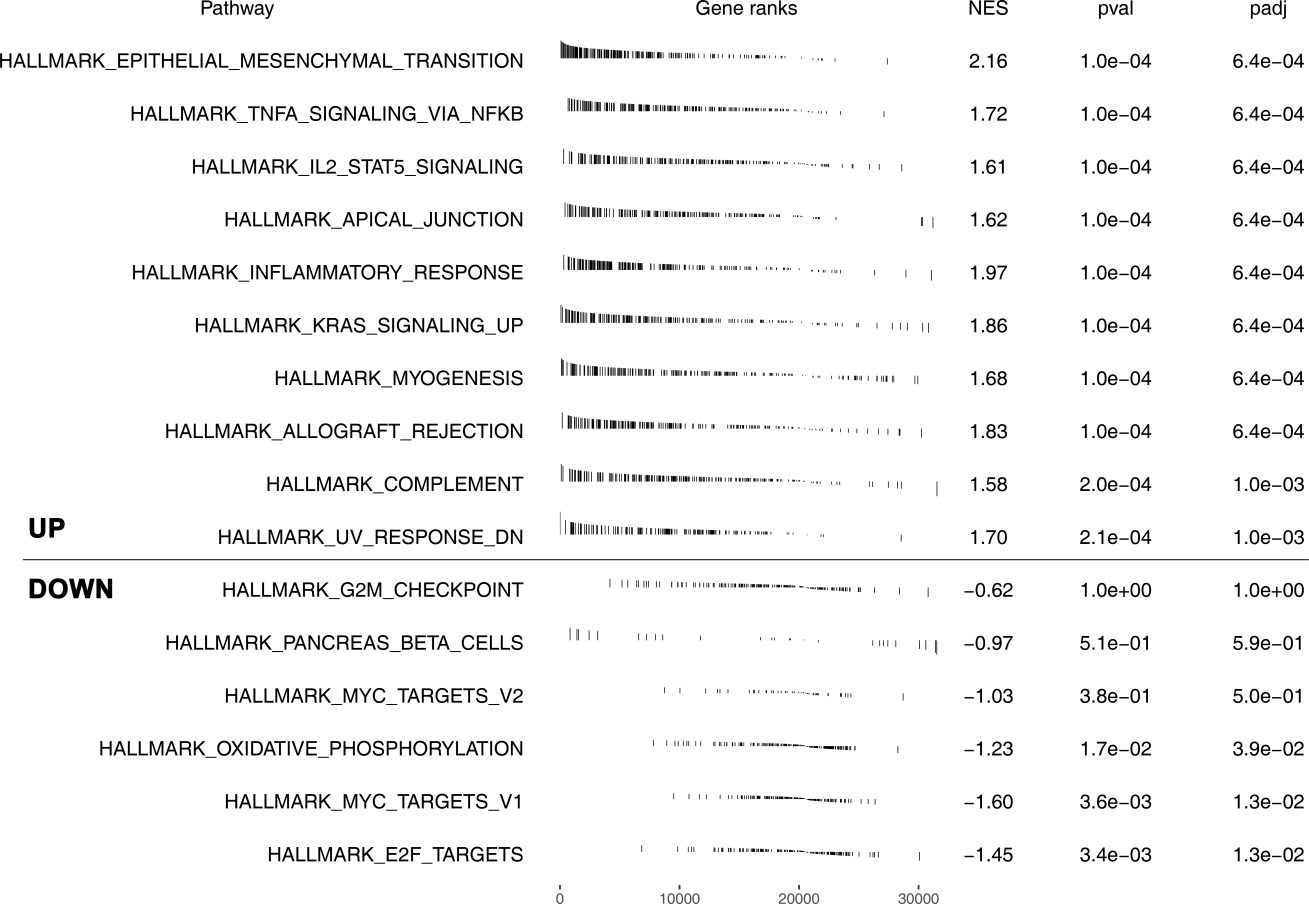
Pathway enrichment analysis of the model.

Further, we explored the clinical correlation of three model genes. The result showed that PTGFR had a lower expression level in tumor tissue, yet LILRA2 and KCNA1 were highly expressed in tumor tissue (**Figure 5A**). Meanwhile, we found that PTGFR and LILRA2 were highly expressed in the patients with lymphatic invasion (**Figure 5B**). Only KCNA1 was observed to have a higher expression level in the older patients (**Figure 5C**). In the patients with different primary therapy outcomes, histologic grade and anatomic neoplasm subdivision, no significant difference was observed in the expression level of PTGFR, LILRA2 and KCNA1 (**Figures 5D–F**). Interestingly, we found that the LILRA2 was highly expressed in the white population compared to other races (**Figure 5G**). Meanwhile, PTGFR, LILRA2 and KCNA1 were all highly expressed in the patients with venous invasion (**Figure 5H**). Pathway enrichment analysis showed that in high risk patients, the pathway of epithelial-mesenchymal transition (EMT), TNF-α signaling *via* NFKB, IL2/STAT5 signaling, apical junction, inflammatory response, KRAS signaling, myogenesis were activated (**Figure 6**). GSEA analysis of GO showed that in high risk patients, the terms of regulation of system process, regulation of vesicle-mediated transport, cell surface receptor signaling pathway involved in cell-cell signaling, regulation of cytoskeleton organization, sensory organ development, cell body, leukocyte differentiation, morphogenesis of epithelium, regulation of lymphocyte activation, regulation of transmembrane transport were activated (**Figure S3A**). GSEA analysis of KEGG showed that in high risk patients, the terms of pathways in cancer, cytokine-cytokine receptor interaction, neuroactive ligand receptor interaction, regulation of actin cytoskeleton, focal adhesion, chemokine signaling pathway, calcium signaling pathway, cell adhesion molecules cams, vascular smooth muscle contraction, ECM receptor interaction were activated (**Figure S3B**).

### Immunotherapy and chemotherapy sensitivity

**Figure 7:**
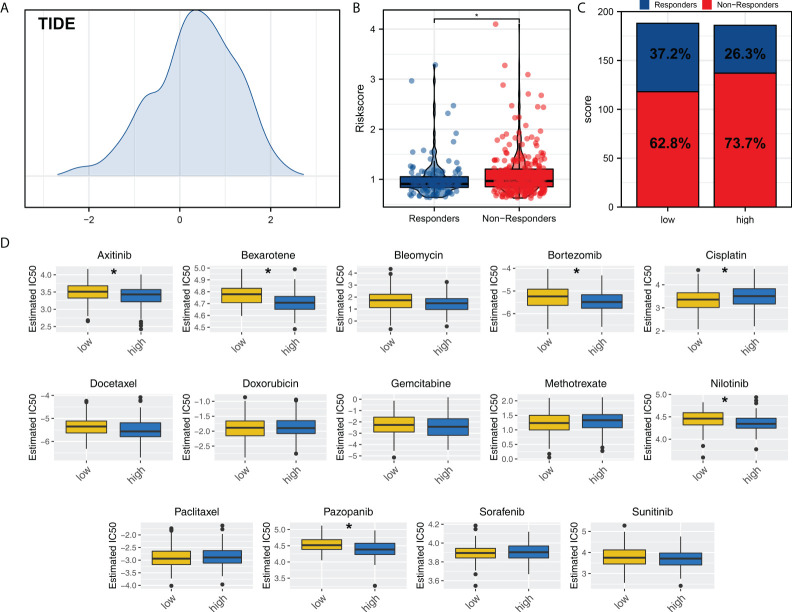
Immunotherapy and chemotherapy sensitivity. **(A)** TIDE analysis was performed to evaluate the immunotherapy response rate of OV patients; **(B)** The riskscore difference of immunotherapy responders and non-responders; **(C)** Patients in low risk group tend to have a higher proportion of immunotherapy responders; **(D)** Chemotherapy sensitivity analysis. *P < 0.05.

We next explored the immunotherapy and chemotherapy sensitivity differences between high and low risk patients. TIDE algorithm was used to evaluate the immunotherapy response rate of OV patients (**Figure 7A**). The result showed that the immunotherapy responders have a lower riskscore (**Figure 7B**). Meanwhile, the low risk group had a higher proportion of immunotherapy responders (**Figure 7C**). Chemotherapy sensitivity analysis showed that the low risk patients might be more sensitive to cisplatin, while high risk patient might be more sensitive to axitinib, bexarotene, bortezomib, nilotinib, pazopanib (**Figure 7D**).

### Further exploration of the PTGFR, LILRA2 and KCNA1

**Figure 8:**
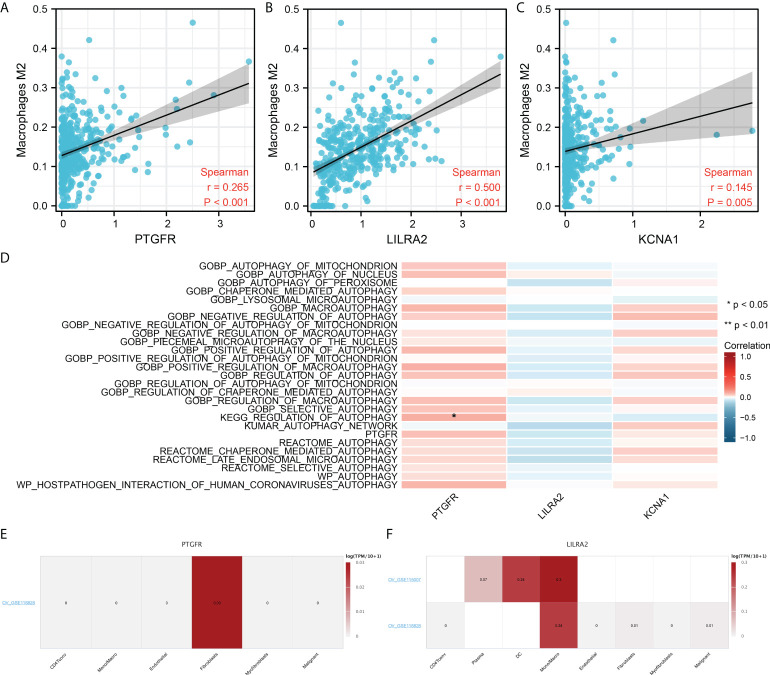
Further exploration of the PTGFR, LILRA2 and KCNA1. **(A–C)** PTGFR, LILRA2 and KCNA1 were significantly positively correlated with the M2 macrophages infiltration; **(D)** The association between model genes and autophage activity; **(E)** PTGFR was mainly expressed in the fibroblasts; **(F)** LILRA2 was mainly expressed in Mono/Macro. *P < 0.05.

We next evaluated the correlation between three model genes PTGFR, LILRA2, KCNA1 and M2 macrophages. The result showed that the PTGFR, LILRA2 and KCNA1 were all positively correlated with M2 macrophages (**Figures 8A–C**, PTGFR, R = 0.265, P < 0.001; LILRA2, R = 0.500, P < 0.001; KCNA1, R = 0.145, P < 0.001). Moreover, we noticed that the PTGFR was significantly associated with the pathway of autophagy regulation (**Figure 8D**). Single-cell analysis showed that the PTGFR was mainly expressed in the Fibroblasts, and LILRA2 was predominantly expressed in the macrophages (**Figures 8E–F**).

## Discussion

OV is a common malignancy all over the world (21). For the insidious symptoms, many OV patients are already in the late stage when they are diagnosed (22). Despite improvements in surgical techniques, the prognosis of patients with OV is not satisfactory for its refractoriness and relapse (22). M2 macrophages have been reported to play an important role in OV development (23). A depth inquiry of biological factors affecting M2 macrophage infiltration could point to novel therapeutic targets.

In our study, we comprehensively analyzed the role of M2 macrophages and M2-macrophages related genes in OV based on the CIBERSORT algorithm. A total of 196 DEGs were identified between OV patients with high and low M2 macrophage infiltration, which were defined as M2 macrophage related genes. PPI network was constructed to explore the underlying interaction between these M2 macrophages related genes. Finally, a prognosis model based on the PTGFR, LILRA2 and KCNA1 was established, which demonstrated a good prediction efficiency in both training and validation cohorts. The result showed that the patients in high risk might have a worse prognosis. A nomogram was constructed for a better clinical application in the clinical. Clinical correlation and pathway enrichment analysis were then performed to explore the underlying differences between high and low risk groups. Furthermore, we further explored PTGFR, LILRA2 and KCNA1 for future studies.

ClueGO analysis showed that the M2 macrophages related genes were mainly enriched in the positive regulation of interferon (IFN)-β biosynthetic process, monoamine transport, phospholipase C-activating G protein-coupled receptor. IFN-β played an important role in regulating M2 macrophage polarization and function (24). Rackov et al. found that the p21 could mediate macrophage reprogramming through regulation of IFN-β, which might be an underlying target for sepsis treatment (25). Kumaran et al. indicated that IFN-β is a macrophage-derived effector cytokine facilitating the resolution of bacterial inflammation through STAT3 signaling (26). Moreover, Xu et al. found that the sustained production of IFN-β could inhibit the OV growth through the macrophage-inducible nitric oxide synthase effect (27). Meanwhile, Trauelsen et al. found that extracellular succinate hyperpolarizes M2 macrophages through SUCNR1/GPR91-mediated Gq signaling (28).

Our model identified three model genes, including PTGFR, LILRA2 and KCNA1. PTGFR (prostaglandin F receptor) is a member of the G-protein coupled receptor family, which is the receptor of prostaglandin F2-alpha (29). Anderson et al. found that the PTGFR is an underlying biomarker for the early detection of OV through the detection of serum antigen (30). LILRA2 (leukocyte immunoglobulin-like receptor, subfamily A, member 2) is a member of a family of immunoreceptors that are expressed predominantly on monocytes and B cells (31). Lu et al. found that the LILRA2 could selectively modulate LPS-mediated cytokine production and hamper phagocytosis by monocytes (32). KCNA1 (potassium channel, voltage-gated shaker related subfamily A, member 1) encodes a voltage-gated delayed potassium channel that is phylogenetically related to the Drosophila Shaker channel (33). Mariani et al. revealed that KCNA1 is an important gene involved in the intestinal metastasis process of OV (34). However, little research was found between these three genes and M2 macrophages in OV. Our results might provide a novel direction for PTGFR, LILRA2 and KCNA1 in OV development.

Pathway enrichment analysis showed that in high risk group, the pathway of EMT, TNF-α signaling *via* NFKB, IL2/STAT5 signaling, apical junction, inflammatory response, KRAS signaling, and myogenesis were activated. EMT pathway exerts an important role in cancer development (35). Liu et al. found that TRPM7 could facilitate the EMT pathway in ovarian cancer through the calcium-related PI3K/AKT oncogenic signaling (36). Deng et al. revealed that targeting EMT and cancer stem cell might be helpful for the treatment of chemoresistant OV (37). Thaklaewphan et al. found that Kaempferia parviflora extract could hamper the TNF-α-induced release of MCP-1 in OV through the suppression of NF-κB signaling, further suppressing the OV development (38). Wu et al. indicated that the STAT3/STAT5 signaling might be an emerging therapy choice for OV (39).

Though our result was based on the high quality of analysis, some limitation should be noticed. Firstly, the population included in our analysis was predominantly white population, which might bring underlying race bias for the application of our conclusions to other races. Secondly, though the prognosis information of OV patients can be obtained, however, the clinical stage of most patients is unknown. If all the clinical features of patients can be obtained, our conclusion might be more stable.

## Data availability statement

Publicly available datasets were analyzed in this study. This data can be found here: https://portal.gdc.cancer.gov/; https://www.ncbi.nlm.nih.gov/gds/?term=.

## Author contributions

CP performed all the bioinformatic analysis. CP and LL wrote the manuscript. All authors contributed to the article and approved the submitted version.

## Conflict of interest

The authors declare that the research was conducted in the absence of any commercial or financial relationships that could be construed as a potential conflict of interest.

## Publisher’s note

All claims expressed in this article are solely those of the authors and do not necessarily represent those of their affiliated organizations, or those of the publisher, the editors and the reviewers. Any product that may be evaluated in this article, or claim that may be made by its manufacturer, is not guaranteed or endorsed by the publisher.

## Supplementary material

Supplementary Figure 1 The flow chart of the whole study.

Supplementary Figure 2 The correlation of M2 macrophages and other immune cells.

Supplementary Figure 3 GO and KEGG analysis based on the GSEA algorithm. **(A)** GO analysis based on the GSEA algorithm; **(B)** KEGG analysis based on the GSEA algorithm.
