# Supplementary figures and images for: Integrated analysis of the M2 macrophage-related signature associated with prognosis in ovarian cancer
Source: Front Oncol. 2022 Aug 26;12:986885. doi: 10.3389/fonc.2022.986885 (PMC9458878; doi:10.3389/fonc.2022.986885)

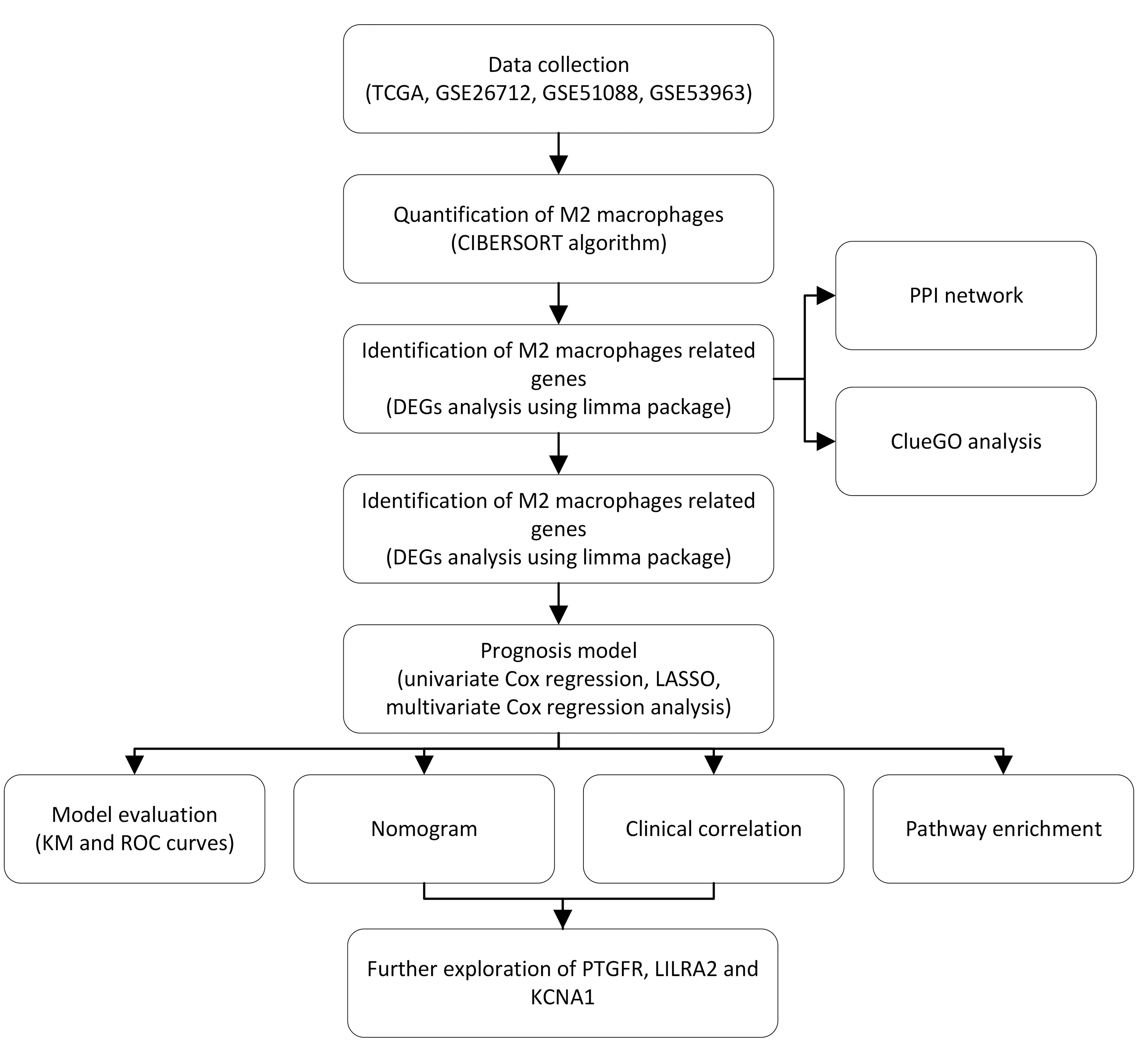

Supplement: Supplementary Figure 1 — The flow chart of the whole study. [file Image_1.tif]

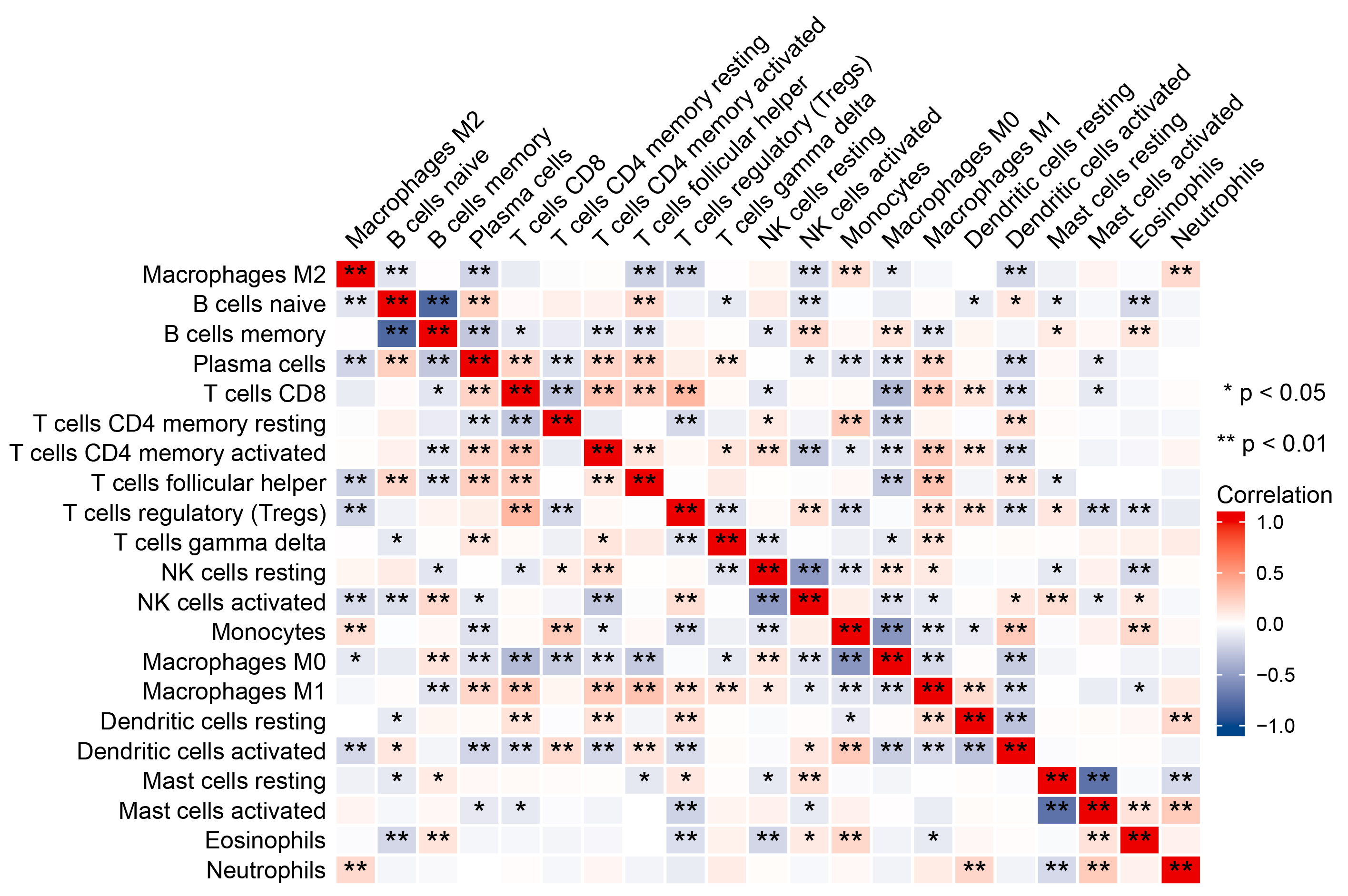

Supplement: Supplementary Figure 2 — The correlation of M2 macrophages and other immune cells. [file Image_2.tif]

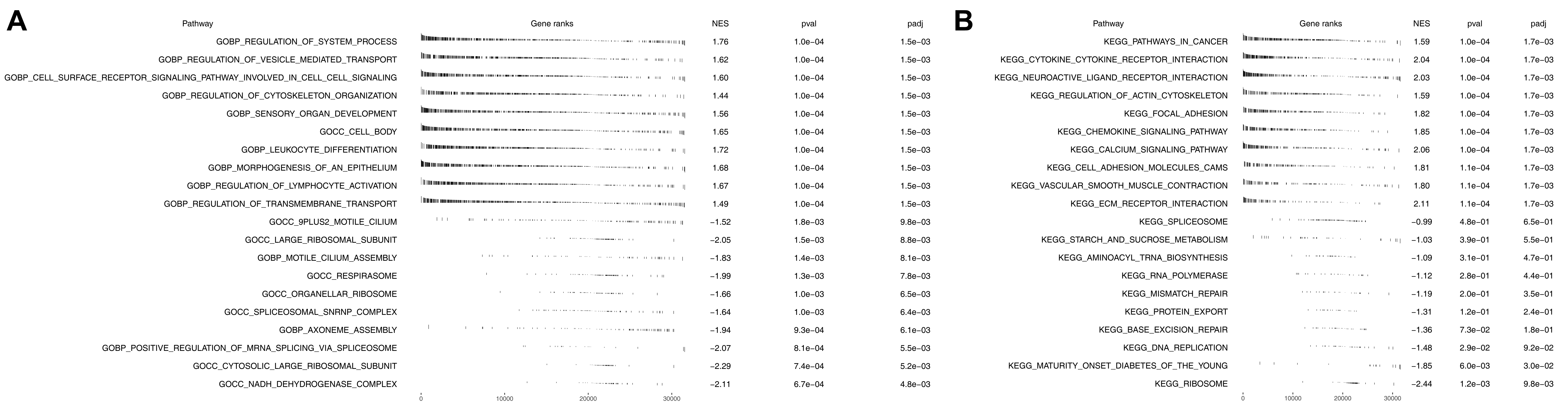

Supplement: Supplementary Figure 3 — GO and KEGG analysis based on the GSEA algorithm. (A) GO analysis based on the GSEA algorithm; (B) KEGG analysis based on the GSEA algorithm. [file Image_3.tif]
